# Creative expressive therapy in schools to address childhood trauma: a mixed methods study of a whole school trauma-informed approach

**DOI:** 10.3389/frhs.2026.1818783

**Published:** 2026-06-22

**Authors:** Lucinda Grummitt, Kirsten Rowlinson, Ben Rockett, Louise Birrell, Rita Daher, Natalie Mackenzie, Nicola C. Newton, Emma L. Barrett

**Affiliations:** 1 The Matilda Centre for Research in Mental Health and Substance Use, The University of Sydney, Sydney, NSW, Australia; 2 KidsXpress Ltd, Macquarie Park, NSW, Australia

**Keywords:** childhood trauma, creative-expressive therapy, implementation science, school-based intervention, trauma-informed practice

## Abstract

**Introduction:**

Schools offer unique opportunities to deliver mental health support for students impacted by trauma. The current mixed-methods study explored the implementation and early effectiveness of the KidsXpress School Partnership Program, a school-based partnership that embeds creative-expressive therapists and trauma-informed consultants within primary schools to deliver targeted therapy for students and whole-school capacity-building for staff.

**Methods:**

Five primary schools in Australia participated. Implementation was assessed via online surveys and interviews from schoolteachers, KidsXpress therapists and trauma-informed consultants. Thematic analysis with a hybrid inductive–deductive approach was used, guided by the Reach, Effectiveness, Adoption, Implementation, and Maintenance (RE-AIM) framework. Data was collected from students via routine assessments as part of the School Partnership Program. Effectiveness was evaluated via changes in wellbeing scores among students referred for creative-expressive therapy sessions from first to last therapy assessment using the Child Outcome Rating Scale (CORS), as well as quantitative and qualitative data from teachers, therapists and trauma-informed consultants.

**Results:**

87 school staff and seven KidsXpress staff participated in online surveys. Nine participants were interviewed, including six KidsXpress staff and three school staff members. Pre-post data on the CORS was available for 233 students. Feedback from teachers and KidsXpress staff indicated strong perceived student benefits, including enhanced emotional regulation and coping, increased confidence, and greater help-seeking. This was supported by wellbeing scores, which were significantly higher at post-assessment compared to first assessment. Teachers reported increased understanding of trauma-informed practice, classroom strategies, and valuable mentoring from KidsXpress staff. The most frequently cited challenges were insufficient staffing and cultural differences in perceptions of mental health. While participants generally felt that trauma-informed practice had improved since the program's introduction, confidence in sustaining these changes without ongoing support was low.

**Conclusions:**

The KidsXpress School Partnership Program is effective in improving student wellbeing following exposure to traumatic experiences and in improving trauma-informed practice at the whole of school level. Nonetheless, implementation barriers exist, and low confidence, early in the partnerships, in the maintenance of trauma-informed practice underscores the need for strategies to embed trauma-informed approaches into school systems to ensure continuity.

## 1 Background

Globally, one in two children are exposed to trauma, such as abuse, neglect, physical and sexual violence, interpersonal loss, or life-threatening illness, before their 18th birthday (1, 2). Trauma exposure at a young age has been linked to substantial immediate and long-term harms, including academic difficulties, alterations in emotional regulation, cognition and interpersonal functioning, heightened health risk factors (e.g., smoking, illicit drug use), mental health and substance use disorders, and chronic disease in adulthood (cancer, cardiovascular disease, diabetes, respiratory disease) (3–6). Indeed, at a population level, childhood abuse and neglect explain between 20%–40% of adult mental health conditions, including mood disorders, substance use disorders, and suicide attempts (7). It is therefore critical to address the impacts of trauma early to ameliorate trauma symptomology and prevent the development of related comorbidities and harms later in life.

Schools are an important setting to address trauma and support the mental health needs of children, with the potential for wide reach and the highest likelihood of reaching youth in need (8). Children exposed to trauma often present in the classroom with difficulties that extend beyond internal distress to include observable challenges in emotional regulation, attention, behaviour, and peer relationships (9). At the primary school level, this may manifest as heightened reactivity, withdrawal, difficulty trusting adults, inconsistent engagement in learning, or behaviours that are perceived as oppositional or disruptive (9). Teachers frequently report feeling underprepared to respond to these complex needs, particularly when behaviours are driven by underlying stress responses rather than intentional misconduct (10). These day-to-day classroom realities highlight the importance of trauma-informed approaches that support not only individual students, but also the educators and systems surrounding them, equipping schools to respond in ways that prioritise safety, co-regulation, and relational support (11). Importantly, addressing trauma within primary school allows for intervention to occur early, before these problems become entrenched and have long-lasting impacts on the health and educational attainment of individuals. Accordingly, there is an increasing provision of trauma-informed and trauma-specific approaches in schools internationally (9, 12–14). The pedagogy for how teachers manage behavioural or academic problems in the classroom are beginning to shift, as schools are increasingly recognising these problems as symptoms of trauma (15) or other underlying difficulties. Indeed, where behavioural outbursts may previously have been addressed with punitive or restrictive behavioural measures, a trauma-informed lens focuses on building the emotion regulation skills and relational capacity of the child instead (15). This shift towards trauma-informed care has resulted in the demand for school-based programs that address the specific needs of trauma-affected children and the unique climate of the individual school (14, 16).

Systematic reviews have identified many trauma programs showing promising results in supporting student mental health, social-emotional wellbeing, and academic outcomes (13, 17). These programs are typically targeted interventions designed to treat trauma symptoms in children who have been exposed to adversity. Cognitive Behavioural Therapy (CBT) is often the primary therapeutic strategy used, with well-established models such as Trauma-Focused Cognitive Behavioural Therapy (TF-CBT) and Cognitive Behavioural Intervention for Trauma in Schools (CBITS) showing positive outcomes (18–20). However, these programs can be cognitively demanding, relying heavily on higher-order executive functioning, which may be challenging for younger children (21), and result in high dropout-rates (22). In response, creative-expressive therapies have emerged as engaging, developmentally appropriate alternatives that may enhance participation and accessibility for children. These modalities, such as art, music, and play therapy, are increasingly being integrated into school-based trauma interventions, either as standalone treatments or as supplements to traditional approaches. Recent reviews suggest that creative therapies are associated with significant improvements in trauma-related symptoms and may offer a more flexible and child-centred approach to trauma recovery (23–25).

Despite the empirical evidence supporting the efficacy of school-based, creative expressive interventions to support students exposed to trauma (24, 25), the feasibility and acceptability of implementing these interventions in real-world practice is critically under-researched. Even the most effective interventions have limited value if they cannot be successfully delivered in practice and sustained long-term. In the school setting, principals and teachers are key stakeholders and their support is crucial to sustainable and successful implementation of mental health interventions (26). Successful implementation relies on motivation and behavioural change of school staff, with studies demonstrating that individual implementation barriers such as stakeholder attitudes, dedicated time, and support often predict the success of program adoption (26–29). In the case of school-based programs for students exposed to trauma, the broader climate of the school, including staff attitudes and readiness for trauma-informed care, has been posited to influence the success and sustainability of these interventions, however there is a dearth of research examining this (30, 31). It is crucial to evaluate implementation strategies alongside whole-of-school attitudes toward trauma-informed care to understand how these factors influence successful integration of school-based trauma interventions.

To our knowledge, there is only one study that has investigated the facilitators and barriers in implementing art therapy in the school system (32). Identified challenges included maintaining art therapy sessions alongside inflexible school schedules, limited therapy rooms available, unstructured therapeutic approaches, and a variety of art tools required. Despite these challenges, there was an overall positive reception to the art therapy. Cited facilitators for implementing art therapy include the playful nature of the therapy initiating close bonds between the student and therapist, an atmosphere of openness in the group settings, and its universality for all students to engage with across learning abilities (32).

The current paper describes a mixed-methods study to examine the implementation and effectiveness of a school-based trauma intervention that utilises creative-expressive therapy to support primary school-aged children (the KidsXpress School Partnership program). KidsXpress has been offering trauma-informed creative-expressive therapy for children (4 to 12 years of age) since its establishment in 2005, with the School Partnership Program being their more recently developed program. This program is a multi-target model, consisting of two core components: creative-expressive therapy via individual or group sessions with students referred to therapy due having experienced an adverse challenge or traumatic event; and whole-school implementation of trauma-informed practices, via trauma-informed consultants embedded within the school. Therapy sessions are delivered by KidsXpress creative-expressive therapists, who are trained in modalities such as music, art, play, drama, and dance movement therapy. Trauma-informed consultants provide specialist trauma-informed training and coaching to all teachers within the school. They coordinate psychoeducation courses for school staff and offer pedagogical advice and coaching through the lens of trauma-informed practice. Seminars are available for parents and caregivers to inform and engage key relational networks on the child in the therapy process. This collaborative approach enables a common language of trauma care to be developed and is based on research demonstrating the importance of involving a child's entire care network in the therapeutic support (33). The KidsXpress School Partnership Program was developed through an integrative, practice-led framework that has been informed by multiple psychological and developmental theories, notably trauma-informed care principles, attachment theory, and neurodevelopmental models of trauma, which emphasise safety, relational attunement, and bottom-up regulation strategies (23, 34, 35). These perspectives are closely aligned with contemporary neuroscience research on brain development and stress regulation, which highlights the role of co-regulation in supporting emotional regulation, learning, and adaptive functioning in children exposed to chronic stress (6, 36).

KidsXpress is also informed by ecological systems theory, which conceptualises child development and wellbeing as emerging from the dynamic interactions between individual, relational, and broader socio-cultural contexts. Accordingly, the model integrates developmentally attuned, expressive therapeutic interventions with engagement across the child's surrounding systems in order to support sustained and contextually embedded change. Creative-expressive therapies draw on these theoretical foundations by engaging sensory, emotional, and symbolic processes to support regulation and meaning-making prior to higher-order cognitive integration (23).

### 1.1 Study objectives and hypotheses

The present mixed-methods study aimed to evaluate the implementation and early effectiveness of the KidsXpress School Partnership Program, a whole-school trauma-informed model incorporating targeted creative-expressive therapy for primary school-aged children. Specifically, the study had two primary objectives:

1. Implementation objective: To examine program reach, adoption, implementation processes, and perceived sustainability of the KidsXpress School Partnership Program at the whole-school level.
2. Effectiveness objective: To assess changes in student wellbeing among children referred to and participating in creative-expressive therapy delivered through the program.

Consistent with prior evidence for creative-expressive and trauma-informed interventions (23–25), we hypothesised that students receiving creative-expressive therapy would demonstrate significant improvements in wellbeing from pre- to post-intervention, as measured by the Child Outcome Rating Scale (CORS). Implementation outcomes were descriptive rather than hypothesis driven.

## 2 Methods

### 2.1 Study design

This study employed a convergent mixed-methods design to evaluate both the implementation and effectiveness of the KidsXpress School Partnership Program. Quantitative and qualitative data were collected in parallel from multiple stakeholder groups and integrated during interpretation to provide a comprehensive assessment of program outcomes.

### 2.2 Summary of study variables, participants, and comparisons

Primary implementation outcomes were the reach, adoption, implementation processes, and maintenance indicators, assessed via surveys and interviews with school staff and KidsXpress personnel. The primary effectiveness outcome was student wellbeing, measured using the Child Outcome Rating Scale (CORS), assessed at the first and last therapy session. In the present study, wellbeing was conceptualised as a multidimensional construct reflecting children's subjective sense of personal, relational, and social functioning. This aligns with trauma-informed and developmental perspectives that emphasise emotional regulation, interpersonal safety, and perceived coping as central indicators of recovery following adverse experiences. Accordingly, wellbeing was operationalised using the Child Outcome Rating Scale (CORS), which captures self-reported emotional distress, relationship functioning, and overall life satisfaction in a brief, developmentally appropriate format.

Study groups included students: children referred for and participating in creative-expressive therapy; school staff: teachers and school support staff at participating schools; and KidsXpress staff: creative-expressive therapists and trauma-informed consultants embedded at participating schools.

The following comparisons were assessed: (1) descriptive comparisons of implementation outcomes across stakeholder groups and schools; (2) within-subject pre–post comparisons of student wellbeing (first vs. last CORS assessment); and (3) exploratory analyses examining whether changes in wellbeing differed by therapy modality (group vs. individual), age, sex, or school context.

### 2.3 Participants and setting

Five primary schools in NSW, Australia were included in this study. Based on the Socio-economic Indices for Areas (SEIFA) Index of Relative Socio-economic Advantage and Disadvantage (IRSAD), three schools were located in areas with an IRSAD score of 3 and two in areas with a score of 4, indicating that the sample was drawn from communities with slightly below-average to average levels of socioeconomic status (37). Participants included (1) students aged 5–12 years who were referred to the KidsXpress School Partnership Program due to exposure to an adverse challenge or traumatic event, (2) schoolteachers and support staff, and (3) KidsXpress creative-expressive therapists and trauma-informed consultants embedded within participating schools.

For students, age at commencement and sex at birth were recorded as part of routine KidsXpress intake assessments. Across the five schools, pre–post wellbeing data were available for 233 students (mean age=8.8 years, SD = 1.8; 52.4% male).

### 2.4 Recruitment and consent

A letter outlining the aims of the research trial was emailed to the school principal of schools enrolled in the School Partnership program. This letter provided information on what would be required of their school if they agreed to participate, including commitments, time frames, and data measures. Once school principals agreed to participate in the study, written consent was sought individually for teachers and KidsXpress staff to participate in the surveys and qualitative interviews. Participation in the research was voluntary and participation in the School Partnership Program was not affected for those who chose not to participate in the research.

Teachers and the KidsXpress team within the school identify specific children and refer them to therapy using a standardised referral form. Parents/caregivers may also refer their child to the program. Any child who is identified as having experienced an adverse challenge, trauma or lifetime adverse event may be referred, such as out-of-home-care, bereavement, bullying/peer relationship difficulties, chronic illness/disability, domestic violence, history of abuse/neglect, parental separation, self-esteem/anxiety issues, or parent substance misuse. To participate, students must have written consent from a caregiver. Children's willingness to participate in the program was determined through a verbal, developmentally appropriate assent process, which was revisited throughout their engagement.

Prior written consent has been obtained from caregivers for their child’s de-identified data to be used for research pertaining to project evaluation and quality improvement purposes.

### 2.5 Intervention

The KidsXpress School Partnership Program is a multi-target, collaborative approach to addressing trauma in primary school-aged children. The Partnership lasts for a minimum of three years, with schools in the current study at varying points in the partnership, ranging from the initial year of establishment to the final year. The Partnership involves a team of three KidsXpress staff (two therapists and one trauma-informed consultant) who are embedded part-time within the school (three days per week each). The team may have representation in a school over more than three days per week, though each individual staff member only works three days each week. Therapists deliver individual and group therapy for students, while the trauma-informed consultant works at the whole-school level to provide psychoeducation for school staff and caregivers, alongside coaching, mentoring, and role modelling of trauma-informed and responsive approaches with children and whole class support. Educators and caregivers are provided with supplementary resources to accompany training, and may seek out KidsXpress staff embedded within the school, to aid in the application of trauma-informed approaches and to address any personal or vicarious distress arising from program engagement. This design is intended to promote a consistent and sustainable shift in school cultural attitudes towards trauma-informed care. The program is funded through financial contribution of the schools involved in the partnership as well as philanthropic donors.

Eligible students identified through school referral processes received individual or group creative-expressive therapy as part of the School Partnership Program. Therapists make a clinical assessment, considering factors such as trauma history and current crisis state to guide the clinician's approach to therapy goals and the therapeutic alliance formation. The program offers a combination of individual (60 min) and group (90 min) therapy sessions, with up to 6 students per group. Children engage in weekly therapy in line with the school term. This can range from some children accessing a term of support (10 weeks) to other children accessing multiple terms. Group therapy sessions generally run across two school terms (20 weeks), with individual therapy generally consisting of up to 25–35 sessions, depending on the needs of the child and therapeutic goals. KidsXpress base their therapeutic approach on Malchiodi's model of creative arts therapy as an approach for trauma resolution (23). It is designed to improve mental health and social-emotional wellbeing by equipping children with the means to better understand, regulate, and express their emotions related to past trauma experience. In contrast to trauma-informed cognitive-behavioural therapy, creative-expressive therapy's core features are sensory and symbolic, consisting of a bottom-up approach that engages body-based, sensory and emotional processes before verbal and cognitive processes that involve conscious thought and cognitive insight. It emphasises safety, relational attunement and regulation through play and expression. Creative expressive therapy provides children with a safe space to express and process their experiences through metaphor, play, and imagination, helping them regulate emotions and build personal meaning without needing to verbally retell events.

The School Partnership Program is a whole-school approach, which, in addition to providing creative-expressive therapy, addresses the school culture by embedding a trauma-informed consultant in school three days a week. Trauma-informed consultants are trained in social work or youth work and provide formal and informal coaching and psychoeducation for school staff. Trauma-informed consultants work with all the teachers across the whole school. Alongside the school executives, they may identify particular teachers who need more intensive coaching support. Psychoeducation in the school setting and online seminars are provided to caregivers. The multi-target model of involving the school, parents and caregivers in the therapeutic process bolsters trauma-informed support across various aspects of the child's life, offering a holistic approach to building capacity of the child's caregiving network rather than working with a child in isolation.

### 2.6 Measures

This study combines the Reach, Effectiveness, Adoption, Implementation, and Maintenance (RE-AIM) framework (38) with the Consolidated Framework for Implementation Research (CFIR) (39) to assess implementation and effectiveness of the KidsXpress School Partnership Program. The RE-AIM framework was designed to inform translation of evidence-based programs into practice and heighten public health impact by examining impact on domains beyond clinical outcomes. Reach pertains to the number of individuals who engage with the intervention. Effectiveness evaluates the intervention's influence on key outcomes, including any unintended consequences, improvements in quality of life, and cost-related implications. Adoption addresses the extent and variety of settings and personnel that choose to implement the program and the acceptability of the program within that setting. Implementation examines how well facilitators can pursue the prescribed procedures or therapeutic goals when implementing a program in real-world settings. Lastly, maintenance assesses how well the intervention is sustained over time and integrated into standard practice. The CFIR framework allows for qualitative analysis of the contextual factors that influence the RE-AIM outcomes. The CFIR describes five domains that can be used to systematically assess and articulate contextual factors that may influence a program implementation: 1) Intervention characteristics (e.g., adaptability); 2) outer setting (e.g., external policies and incentives); 3) inner setting (e.g., leadership engagement); 4) individual characteristics (e.g., self-efficacy); and 5) process (e.g., planning) (39).

The RE-AIM framework was selected due to its extensive use in evaluating the real-world impact of school-based and public health interventions, particularly those targeting mental health outcomes (40). RE-AIM provides a structured approach to assessing not only effectiveness, but also reach, adoption, implementation processes, and sustainability, which are critical considerations for interventions delivered within complex school systems. The Consolidated Framework for Implementation Research (CFIR) was used to complement RE-AIM by providing a theoretically grounded structure for analysing contextual influences on implementation. CFIR has been widely applied across health and education settings to enhance the rigor and transparency of qualitative implementation analyses (41). While RE-AIM and CFIR are not psychometric instruments, their validity is supported through extensive empirical use, conceptual clarity, and demonstrated utility in guiding systematic implementation evaluation (40, 41). Together, these frameworks strengthened the analytic rigor of the study by ensuring that both outcomes and contextual determinants of implementation were examined in a comprehensive and theoretically-informed manner.

#### 2.6.1 Data collection instruments

Data was sourced from key stakeholders involved in the School Partnership Program, namely via trial-specific surveys and interviews with teachers, KidsXpress therapists and trauma-informed consultants. Data was also collected from students via routine assessments as part of the School Partnership Program. A summary of measures and collection details can be found in Table 1, and further detail is provided below.

**Table 1:** Summary of measures included in the current study.

RE-AIM Domain	Construct assessed	Measure/Data source	Informant	Data type	Timing
Reach	Referral and participation rates	School administrative data; KidsXpress intake records	Schools/KidsXpress	Quantitative	Ongoing
Effectiveness (student)	Wellbeing	Child Outcome Rating Scale (CORS)	Student	Quantitative	First and last therapy session
Effectiveness (perceived)	Perceived student, staff, and parent benefit	Trial-specific survey items (Likert scale)	School staff; KidsXpress staff	Quantitative	Mid–late implementation
Effectiveness (qualitative)	Perceived outcomes and unintended effects	Open-ended survey items; interviews	School staff; KidsXpress staff	Qualitative	Single time point
Adoption	Acceptability and fit	Survey items on suitability and recommendation	School staff; KidsXpress staff	Quantitative	Single time point
Implementation	Barriers, facilitators, resourcing	Surveys and interviews	School staff; KidsXpress staff	Mixed	Single time point
Maintenance	Perceived sustainability	Survey items and open-ended responses	School staff; KidsXpress staff	Mixed	Single time point

##### 2.6.1.1 Reach

Reach reflects the overall participation rate in KidsXpress therapy. That is, i) of the entire student cohort of the school, what proportion were referred? ii) Of those referred, what proportion participated in the intervention? and iii) How many were placed on a waitlist? This data was collected via school administrative data and KidsXpress intake assessments.

##### 2.6.1.2 Effectiveness

Effectiveness was measured through i) school staff, therapist, trauma-informed consultant perspectives, as well as ii) student change on key indicators and iii) whole-of-school change on key indicators.

(i) Staff, therapist, and consultant perspectives: These groups were asked a series of questions on their perceived effectiveness of the program on a 5-point Likert-type scale. Example items included, “*To what extent do you believe the students/school staff/parents benefitted from the KidsXpress program?; How effective do you think the KidsXpress program was in providing students with the skills to better express their emotions?; How would you rate the overall level of engagement among students?;* and *Did you notice any benefits/ negative effects within the school/community that could reasonably be attributed to the program?”*
(ii) Student change on key indicators: The Child Outcome Rating scale (42) was used to assess student change in overall student functioning. The Outcome Rating Scale (ORS) is a four-item measure used to monitor children's progress to therapeutic intervention. The scale measures personal or symptom distress, interpersonal wellbeing, social satisfaction, and overall wellbeing on a visual scale. Scores on the first and last assessment will be taken as pre and post, respectively.
(iii) Whole-of-school change on key indicators: Student attendance in the 12-month period prior to the start of the KidsXpress School Partnership Program, collected via school administrative records, was compared with the following 12-month period to assess change in student attendance. As per school's annual reporting, attendance is based on Semester 1 of the year quoted.

##### 2.6.1.3 Adoption

Adoption was assessed through a series of survey items from i) staff members; ii) therapists and trauma-informed consultants.

(i) Staff were asked whether they would recommend the KidsXpress program to another school, the extent to which they believe the use of the program is consistent with the mission of their school.
(ii) Therapist and trauma-informed consultants were asked about the reception of KidsXpress by school staff and parents, the receptiveness of the school and parents to trauma-informed care and willingness to refer students or approach the KidsXpress team for advice and support.

##### 2.6.1.4 Implementation

Implementation was measured via self-report surveys and interviews of school staff, therapists, and trauma-informed consultants asking about facilitators and barriers to implementation, suggestions for improvement to make involvement in the School Partnership Program easier, and whether resourcing was adequate.

##### 2.6.1.5 Maintenance

Maintenance was assessed through the following items relating to ongoing delivery of the program within the school, asked of school staff, therapists, and trauma-informed consultants: “*Do you think your school has improved in trauma-informed practice since the implementation of the KidsXpress program?; Do you think the school will maintain this improvement in trauma-informed practice when the KidsXpress team withdraws from the school?; To what extent did you feel embedded within the school?; In your opinion, did the school staffs’/parents’ perception of KidsXpress change over time?”*

##### 2.6.1.6 Qualitative focus and analytic objectives

Open-ended survey responses and semi-structured interviews were used to deepen understanding of implementation processes and perceived program effects. Specifically, the qualitative component addressed the following research questions: (1) how do school staff and KidsXpress staff perceive the benefits of the School Partnership Program for students, staff, and families?; (2) what factors facilitate or hinder implementation of the program within the school context?; (3) how do stakeholders perceive the sustainability of trauma-informed practices following program withdrawal?

These qualitative analyses were intended to contextualise and explain quantitative findings rather than introduce additional study objectives.

### 2.7 Statistical analysis

Sample size calculations were based on a school-based, creative arts therapy intervention study (43). A total of 57 students would be required to achieve 80% power and detect pre-post mean differences of 0.3 (*p* = 0.05). Allowing for 15% dropout, the final required sample size was calculated as 66 students. This number was achieved. As observations from school staff, therapists, and trauma-informed consultants use a combination of qualitative and quantitative data, this does not require a formal sample size calculation. However, these expected numbers will provide sufficient in-depth information to meet the aims of this study (44, 45),

Quantitative data was analysed using R. Descriptive analysis was undertaken based on frequency tables, means with their standard deviations and medians. Paired-samples t-test were conducted to compare children's CORS scores at their first and last assessment during KidsXpress therapy. To account for variability in therapy exposure, we conducted a linear regression analysis to examine whether factors such as therapy type (group vs. individual), school, child age and sex were associated with changes in student wellbeing. Qualitative data from open-ended survey responses and interviews was analysed using thematic analysis, following the six-phase approach outlined by Braun & Clarke, 2022. This includes: (1) familiarisation with the data, (2) generating initial codes, (3) searching for themes, (4) reviewing themes, (5) defining and naming themes, and (6) producing the report (46). A hybrid inductive–deductive approach was employed. Simultaneously, inductive coding allowed for the emergence of unanticipated themes grounded in participants’ experiences. Descriptive summaries of key themes are reported, with illustrative quotes to support interpretation.

Ethics approval was obtained from University of Sydney Human Research Ethics committee (HREC) 2024/HE000137. This research was authorised through the NSW Department of Education State Education Research and Partnerships (SERAP) process (2022265). This project was conducted in collaboration with KidsXpress, developers of the program and implementation partners, who provided critical input into the design of the study. The researcher team led data collection, analyses and interpretation and reporting of results.

## 3 Results

### 3.1 Sample

A total of 87 school staff (reflecting an average of 33% of staff at participating schools) from 5 schools participated in online surveys. This was made up of 79 teachers, one Student Support Officer, and seven who indicated “other” for their role. School staff participants were located at School 2 (*n* = 45), School 1 (*n* = 27), School 4 (*n* = 9), School 3 (*n* = 6). No school staff from School 5 participated. In addition, seven KidsXpress staff participated in online surveys. Five were employed as therapists, and two were trauma-informed consultants.

Nine participants were interviewed for this study, including six KidsXpress staff (two trauma-informed consultants, four creative-expressive therapists), and three school staff members. The majority of participants practiced in School 2 (*n* = 5), and the remaining practiced in School 3 (*n* = 2), School 4 (*n* = 1), and School 5 (*n* = 1).

Pre-post data on the CORS was available for 233 students across the five participating schools. Students were 8.8 years old on average (Standard Deviation=1.8), and 52.4% were male.

### 3.2 Reach

Table 2 shows the reach of KidsXpress therapy at the partnership schools. There was substantial variability between schools, with between 7% – 57% of the student cohort referred for therapy. Schools that had a longer time period since establishment showed greater referral rates. The majority of referred students participated in therapy (73% - 98%), with few placed on a waitlist.

**Table 2:** Reach.

School	1	2	3	4	5
Entire student cohort (2024), *N*	399	805	598	317	579
Referrals *N*, %	229 (57.4%)	272 (33.8%)	55 (9.2%)	54 (17.0%)	41 (7.1%)
Participants *N*, % of intake	209 (91.3%)	247 (90.8%)	40 (72.7%)	53 (98.1%)	36 (87.8%)
Waitlist	20	25	15	1	5

### 3.3 Effectiveness

#### 3.3.1 School staff perspectives

Survey responses from school staff are shown in Table 3. Staff were generally very positive about the KidsXpress partnership, with most noticing great benefit for students and school staff, with somewhat lower perceived benefit for parents. Most reported a great improvement in their own, and the school's, awareness of trauma-informed practice.

**Table 3:** School staff survey results (valid *n* = 86).

Survey item	1 (Most negative) *N* (%)	2 *N* (%)	3 *N* (%)	4 *N* (%)	5 (Most positive) *N* (%)
To what extent do you believe the students benefitted from the KidsXpress program?	1 (1.1)	7 (8.0)	18 (20.7)	45 (51.7)	16 (18.4)
To what extent do you believe school staff benefitted from this program?	1 (1.2)	5 (5.8)	17 (19.8)	50 (58.1)	13 (15.1)
To what extent do you believe parents benefitted from this program?	6 (7.0)	17 (19.8)	28 (32.6)	27 (31.4)	8 (9.3)
How effective do you think the KidsXpress program was in providing students with the skills to better express their emotions?	3 (3.5)	5 (5.8)	20 (23.3)	41 (47.7)	17 (19.8)
How would you rate the overall level of engagement among students during the KidsXpress program?	0 (0.0)	1 (1.2)	11 (12.8)	35 (40.7)	39 (45.3)
How useful do you think school staff found the coaching, mentoring, and psychoeducation sessions that were provided by the trauma-informed consultant?	4 (4.7)	2 (2.3)	10 (11.6)	37 (43.0)	33 (38.4)
To what extent do you think your trauma-informed awareness has improved due to KidsXpress?	2 (2.3)	7 (8.1)	18 (20.9)	47 (54.7)	12 (14.0)
To what extent do you think trauma-informed awareness amongst the school has improved due to KidsXpress?	3 (3.5)	5 (5.8)	15 (17.4)	54 (62.8)	9 (10.5)
Did you notice any benefits within the school/community that could reasonably be attributed to the KidsXpress program?	5 (5.9) No		38 (44.7) unsure		42 (49.4) Yes
Did you notice any negative effects within the school/community that could be reasonably attributed to the program?	7 (8.1) Yes				79 (91.9) No
Overall, how useful did you find the KidsXpress program?	0 (0.0)	5 (5.8)	11 (12.8)	29 (33.7)	41 (47.7)

Darker shading reflects a higher frequency of response.

Open-ended survey responses and interviews provided further insights into the perceived benefits of the School Partnership. For students, observed improvements included enhanced emotional regulation and coping strategies, increased confidence, greater willingness to seek help, and the ability to articulate feelings. Teachers also noted a reduction in disruptive behaviours, improved classroom engagement, and a strong sense of safety and trust in the program, indicating that the program provided valuable support to students in need.

“Students are calmer, happier after seeing the therapists.” – School staff member.

“Whatever they do inside their service sessions changes those kids for the better… some of our trickiest, hardest kids have just turned and blossomed into wonderful kids that everyone loves and cherishes.” – School staff member.

Teachers reported benefits for staff such as increased understanding of trauma-informed practice, practical classroom strategies for managing complex behaviours, emotional support and reduced stress through shared responsibility with KidsXpress staff, and valuable mentoring and coaching from KidsXpress staff that enhanced collaboration and whole-school consistency in wellbeing approaches.

“Staff feel supported by KX staff and are able to seek advice.” – School staff member.

Teachers who participated in qualitative interviews reported that the guidance from the School Partnership Program made them reflect on their own practice and become more trauma-informed. They noted that, when they adopted trauma-informed strategies, such as a calm corners or brain breaks, classes seemed more manageable and regulated. Additionally, some teachers reflected that KidsXpress helped them become more compassionate to themselves when they were not perfect in the classroom or were unsure how to help students in the past.

“We’re all aware of trauma-informed practice and how that best supports the kids…Their [KidsXpress’] guidance and strategies and all the learning that they do with us has really made me reflect and be a better teacher”. - School staff member.

Ratings of perceived benefit for parents were somewhat lower, reflecting some challenges in engaging families consistently. Nonetheless, when parents did engage, teachers observed benefits such as reassurance that children could receive specialised support, improvement in home behaviour and parent-child relationships. There was limited but positive feedback on workshops and advice provided to parents.

“Parents can see that their child is being supported and nurtured at school.” - School staff member.

A minority of teachers noted some challenges and negative effects of the partnership program, related to the difficulty engaging parents. They cited some parental resistance or misunderstanding of therapy, which could lead to emotional risks for students without adequate home support, as illustrated by the below.

“Some parents do not want their child referred to KidsXpress as they see it as a negative thing.” - School staff member.

“Not because of KidsXpress being a negative, but I worry for some of our really vulnerable students the wall that is chipped away in the sessions brings up more memories and worries that they had locked up and unfortunately at home they do not have the safety net around them to support their dysregulation.” - School staff member.

#### 3.3.2 Kidsxpress staff perspectives (therapists and trauma-informed consultants)

As shown in Table 4, KidsXpress staff were positive about the effectiveness of the partnership program, with generally greater ratings of benefit than school staff. Particularly for students, they noted a high level of engagement and perceived benefit.

**Table 4:** Kidsxpress staff perspectives on effectiveness.

Survey item	1 (Most negative) *N* (%)	2 *N* (%)	3 *N* (%)	4 *N* (%)	5 (Most positive) *N* (%)
To what extent do you believe the students benefitted from the KidsXpress program?	0 (0.0)	0 (0.0)	0 (0.0)	3 (42.9)	4 (57.1)
To what extent do you believe school staff benefitted from this program?	0 (0.0)	1 (14.3)	3 (42.9)	1 (14.3)	2 (28.6)
To what extent do you believe parents benefitted from this program?	0 (0.0)	0 (0.0)	3 (42.9)	3 (42.9)	1 (14.3)
How would you rate the overall level of engagement among students during the KidsXpress program?	0 (0.0)	0 (0.0)	0 (0.0)	0 (0.0)	7 (100.0)
How would you rate the overall level of engagement among school staff during the KidsXpress program?	0 (0.0)	1 (14.3)	1 (14.3)	4 (57.1)	1 (14.3)
How would you rate the overall level of engagement among parents during the KidsXpress program?	1 (14.3)	2 (28.6)	3 (42.9)	1 (14.3)	0 (0.0)
Did you notice any benefits within the school/community that could reasonably be attributed to the KidsXpress program?	1 (14.3) No				6 (85.7) Yes
Did you notice any negative effects within the school/community that could be reasonably attributed to the program?	3 (42.9) Yes				4 (57.1) No

Shading reflects the frequency of response, with darker shading reflecting a greater frequency of response.

For students, KidsXpress staff noted improved emotional regulation, confidence, and social engagement, as well as access to a safe space and supportive relationships.

“For some children, they don't even have a safe space to be themselves. Our therapy room provides them this opportunity.” – KidsXpress staff member.

KidsXpress staff also observed positive impacts on teachers, such as increased awareness of trauma-informed practices and willingness to seek strategies, though time constraints and competing priorities limited engagement:

“Through building safe and trusting relationships with staff and teachers, there is greater vulnerability and help seeking - particularly with more ‘challenging’ students. This allows for more conversations and training in trauma-informed practice which can help teachers to develop a new lens of connection and co-regulation, which impacts on both the teacher, students and whole classes.” – KidsXpress staff member.

“As we are a new partnership, the teachers are in the early phase of learning about trauma-informed practice, so they are still questioning the approach and seeking behaviour-related improvements in the children.” – KidsXpress staff member.

Perceived parent involvement was mixed, with cultural and language barriers noted as often hindering engagement.

“Parents are often very positive about KidsXpress and the benefits they report at home such as increased happiness/joy, improved mood and emotional regulation, skills for coping and resiliency. However, at our school, it is difficult to engage directly with most parents due to language and cultural barriers, which impacts effectiveness, sustainability and longevity of our program beyond the time of engagement.” – KidsXpress staff member.

#### 3.3.3 Student change on key indicators

**Figure 1:**
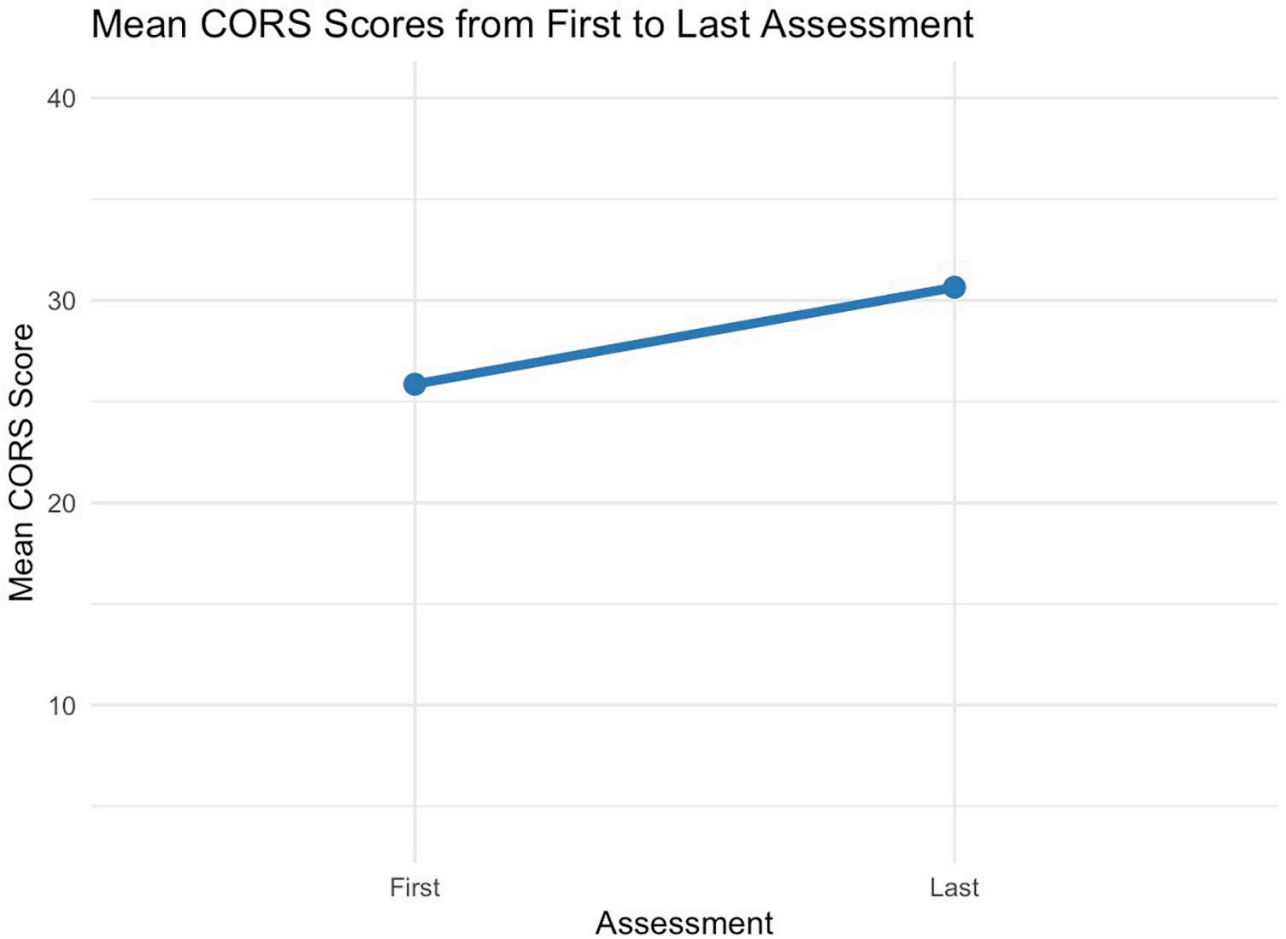
Mean Child Outcome Rating Scale (CORS) scores for students participating in KidsXpress therapy, averaged across all schools, from first to last assessment.

Pre-post CORS data was available for 233 students across the five schools. As shown in Figure 1, scores at the last assessment (M = 30.65, SD = 7.62) were significantly higher than scores at the first assessment (M = 25.86, SD = 8.53), t(232) = 7.23, *p* < .001, two-tailed. The mean difference was 4.79 [95% CI (3.48, 6.09)], indicating that overall, children reported significantly greater wellbeing at the end of the program compared to the beginning.

Two linear mixed-effects models were run. The first model examined overall changes in student wellbeing scores from the first to the last assessment, controlling for age, sex, program type, and school and accounting for repeated measures within children. Results showed a significant improvement of 4.84 points on the CORS at the last assessment compared to the first (*p* < .001), controlling for these other factors. This indicates that, on average, students experienced meaningful gains in wellbeing during their time in the KidsXpress program. Age was negatively associated with wellbeing scores, suggesting older children reported slightly lower scores overall. School-level differences were also evident, with School 2 (Estimate=3.67, *p* < .001) and School 5 (Estimate=6.14, *p* < .001) showing higher baseline scores than School 1. Child sex and program type did not significantly influence outcomes.

**Figure 2:**
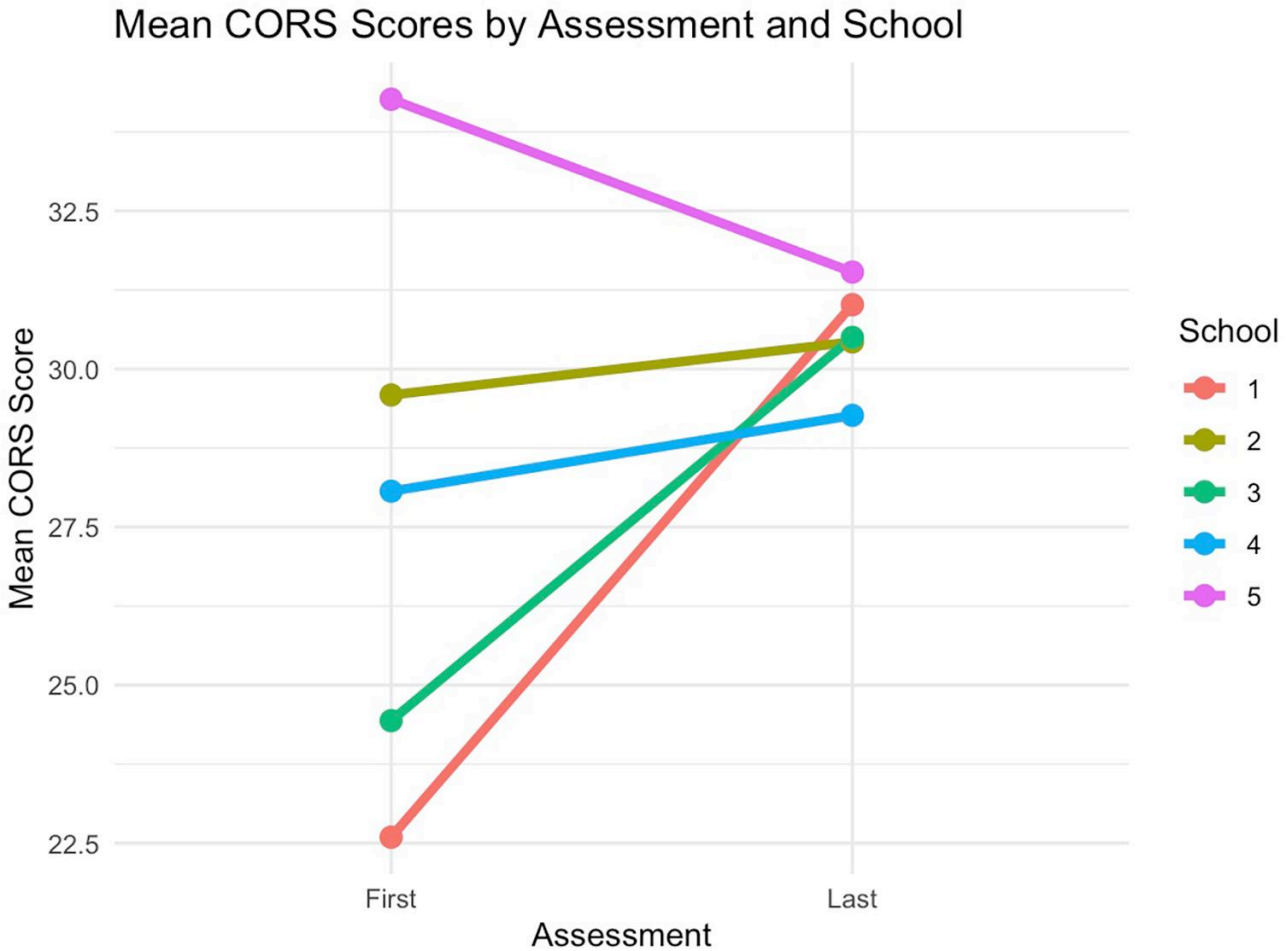
Change in CORS scores from first to last assessment, by school. As shown, schools 1 and 3 show steep increases, compared to the other schools, yet these schools also show the lowest scores at baseline. In contrast, school 5 scores at baseline are almost at the maximum on the CORS (possible maximum is 40), giving them less room for quantitative improvement in mean scores.

The second model explored whether improvements in wellbeing varied by demographic factors or school context by including interaction terms. This model revealed that improvement in CORS scores was not uniform across all groups. Instead, school-level interactions were highly significant: compared to School 1, Schools 2, 4, and 5 showed significantly smaller improvements on CORS scores, while School 3 did not differ significantly. This suggests that while overall wellbeing improved across the program, the magnitude of change varied considerably by school context. However, as shown in Figure 2, these schools had higher baseline scores, and thus had less opportunity to show equivalent gains. Neither sex, age, nor program type significantly moderated the change in CORS scores, indicating that improvements were similar for boys and girls and for individual vs. group therapy, and regardless of age.

#### 3.3.4 Whole of school change of key indicators

 Table 5 shows student attendance in the 12-month period prior to the school's establishment of the Partnership Program compared with the following 12-month period. As shown, four out of five partnership schools saw small increases in attendance, while one saw a small decline.

**Table 5:** School attendance.

Attendance metric	1	2	3	4	5
Attendance year prior	2022: 77.7%	2018: 90.1%	2024: 87.3%	2024: 88.6%	2024: 87.6%
Attendance year following	2023: 82.2%	2019: 87.9%	2025: 89.3%^a^	2025: 91.1%^a^	2025: 88%^a^

a Data reported by principal (annual report not yet released).

### 3.4 Adoption and acceptability

#### 3.4.1 School staff perspectives

The vast majority of school staff (90.5%, *n* = 76) said they would recommend KidsXpress to another school and felt it was consistent with the mission of their school (84.5%, *n* = 71). There was also strong belief that school was the best setting for the KidsXpress therapy (83.3% yes, 14.3% unsure, 2.4% no). As shown in Table 6, school staff rated the suitability of the School Partnership Program highly.

**Table 6:** Suitability ratings from school staff.

Survey item	1 (Most negative) *N* (%)	2 *N* (%)	3 *N* (%)	4 *N* (%)	5 (Most positive) *N* (%)
To what extent does the KidsXpress program seem suitable in addressing the needs of children who have experienced trauma?	0 (0.0)	2 (2.4)	12 (14.1)	48 (56.5)	23 (27.1)
To what extent does the KidsXpress program seem suitable in providing trauma-informed assistance to school staff?	3 (3.5)	4 (4.7)	11 (12.9)	41 (48.2)	26 (30.6)

Shading reflects the frequency of response, with darker shading reflecting a greater frequency of response.

Open-ended survey responses revealed a number of strengths to the KidsXpress approach. Common themes included the creative, arts-based approach being highly valued for engaging students, embedded support within schools worked well to build trust and enable real-time collaboration, the professionalism and expertise of KidsXpress team was consistently praised, with staff liking the capacity building opportunities and guidance. The educator role was seen as critical for embedding strategies across classrooms.

“The therapists are very professional, calm and friendly. The students love going to the KidsXpress sessions!” – School staff member.

“Being embedded within the school so that school staff and families felt KidsXpress was part of the school community. The extended partnership is absolutely necessary to gain the trust of families in this school context.” – School staff member.

While over one-third of school staff indicated there was nothing that could have been done better (*n* = 31, 36.5%), open-ended survey responses suggested areas for improvement including communication (providing more frequent updates to teachers on student progress and strategies); capacity (increase staffing to meet demand; extend support to younger students); and collaboration (strengthen integration with classroom practice and leadership teams).

“Email teachers frequently regarding strategies used with students. Do not wait to give a summary report of the student.” – School staff member.

#### 3.4.2 Kidsxpress staff perspectives

Therapist and trauma-informed consultants felt they were received positively by the school, as shown in Table 7, though there was some ambivalence in how well they felt embedded in the school. KidsXpress staff were split in terms of whether they felt parents were willing to approach a member of the team for advice or support, and the receptiveness of parents to trauma-informed care.

**Table 7:** Kidsxpress staff perspectives on their reception within schools.

KX staff	1 (Most negative) *N* (%)	2 *N* (%)	3 *N* (%)	4 *N* (%)	5 (Most positive) *N* (%)
How would you describe the reception of the KidsXpress team by school staff?	0 (0.0)	0 (0.0)	1 (14.3)	4 (57.1)	2 (28.6)
How would you describe the reception of the KidsXpress team by parents?	0 (0.0)	0 (0.0)	2 (28.6)	2 (28.6)	3 (42.9)
Were school staff willing to refer children to the KidsXpress team?	0 (0.0)	0 (0.0)	1 (14.3) Unsure	0 (0.0)	6 (85.7)
Were school staff willing to approach a member of the KX team for advice or support?	2 (28.6) No		0 (0.0)		5 (71.4) Yes
Were parents willing to approach a member of the KX team for advice or support?	3 (42.9) No		1 (14.3) Unsure		3 (42.9) Yes
To what extent were school staff receptive to learning about trauma-informed care?	0 (0.0)	1 (14.3)	2 (28.6)	3 (42.9)	1 (14.3)
To what extent were parents receptive to learning about trauma-informed care?	2 (28.6)	1 (14.3)	2 (28.6)	2 (28.6)	0 (0.0)
To what extent did you feel embedded within the school?	0 (0.0)	1 (14.3)	3 (42.9)	2 (28.6)	1 (14.3)
Do you believe this program can easily fit within most school practices?	1 (14.3)				6 (85.7)
Do you believe this program is disruptive to daily activities or other students?	1 (14.3)				6 (85.7)

Shading reflects the frequency of response, with darker shading reflecting a greater frequency of response.

KidsXpress staff felt school staff were generally willing to refer students, with free text responses indicating they were more likely to make referrals if they had a better understanding of the program.

“Once we had a shared understanding with staff of what the KX program was they were willing to refer students.” – KidsXpress staff member.

“Some teachers are more likely to refer because they understand the program better and have seen the results with previous students.” – KidsXpress staff member.

KidsXpress staff mostly found school staff were willing to approach them for advice, although open-ended survey responses revealed some challenges with engaging school staff and the importance of building relationship and getting to know the school staff. KidsXpress staff felt parents were harder to engage, noting language and cultural barriers. They were generally confident that the partnership program could be applied in most school contexts, however noted that this would be dependent on buy-in from the school executive and the school's receptiveness to the idea of trauma-informed practice and culture. One participant noted disruptions to daily activities and other students, citing that:

“Students miss class time for therapy and when they leave class other students who want to come to KX get a bit distracted and sad.” – KidsXpress staff member.

### 3.5 Implementation

#### 3.5.1 School staff perspectives

Approximately one third of teachers felt the number of KidsXpress staff embedded at their school was insufficient (*n* = 26, 30.6%), while 16.5% (*n* = 14) were unsure. The open-ended responses all called strongly for additional therapists and extended program days to meet high levels of need. Most staff reported that the program was easy to integrate into daily activities and teaching, with nearly three quarters indicating it was “greatly” (*n* = 41, 48.8%) or “completely” (*n* = 21, 25.0%) easy to integrate. In contrast, the majority did not find the program disruptive to daily activities or other students, with 36.9% (*n* = 31) saying “not at all” and 33.3% (*n* = 28) saying “slightly” disruptive.

#### 3.5.2 Kidsxpress staff perspectives

Staff emphasised the importance of relationship building, clear communication, and strong school leadership for success. They valued the program's embedded model, flexibility, and whole-school approach.

The majority of KidsXpress staff (*n* = 5, 71.4%) did not believe the number of KidsXpress staff embedded in their school was sufficient (Table 8).

**Table 8:** Implementation perspectives of kidsXpress staff.

Survey item	Not at all *N* (%)	Slightly *N* (%)	Somewhat *N* (%)	Greatly *N* (%)	Completely *N* (%)
To what extent were therapy session lengths suitable for covering all the desired content?	0 (0.0)	1 (14.3)	1 (14.3)	4 (57.1)	1 (14.3)
How confident do you feel in your ability to implement the Whole School Partnership Program?	0 (0.0)	1 (14.3)	2 (28.6)	4 (57.1)	0 (0.0)
How difficult or easy did you find adhering to session plans?	0 (0.0)	0 (0.0)	6 (85.7)	1 (14.3)	0 (0.0)
Do you think the number of KX staff embedded in your school was suitable?	5 (71.4) No				1 (14.3) Yes

Shading reflects the frequency of response, with darker shading reflecting a greater frequency of response.

“We are 2 therapists in a low-SES-school of 650 students. It feels like we will never get to see every child who could benefit from KidsXpress.”

#### 3.5.3 Facilitators of successful program implementation

In-depth perspectives sourced from interviews revealed several facilitators of successful program implementation**.**

It was often cited that support from the school executive team was integral to the implementation of the School Partnership Program. Staff commented that having the executive support creates an environment where teachers are encouraged to explore trauma-informed approaches in the classroom. School executive members who are passionate and had baseline values that aligned with KidsXpress were considered important facilitators to the success of the program. Further, some participants reflected that in order to sustain future trauma-informed practice within the school once KidsXpress has left, support needs to occur from the top down.

“The executive team are really on board and they themselves are quite trauma-informed in their approaches and are really passionate…The baseline values are very much aligned, which I think is really important for the success of the program”. – KidsXpress staff member.

Many participants reported the importance of establishing a foundational relationship between KidsXpress and the school community, before meaningful support can take place. KidsXpress therapists commented that building a network of safety and trust with students and teachers is required before they can provide therapeutic sessions. Similarly, trauma-informed consultants commented that, not only is it necessary for them to observe and understand the current practices in school, but teachers also appreciated when consultants acknowledged their efforts in consultations. School staff commented that, by taking the time to build a relationship, the KidsXpress team become more accepted within the school community.

“I really value the environments of relationship building within the school. The beauty of being embedded in a school is that I get to actually know the teachers, the school staff, the office lady, the canteen lady, the students at the school…I believe that's half the work”. – KidsXpress Staff member.

Participants reported that having school staff members who champion trauma-informed teaching assists in program implementation and shifting school culture. Buy-in from school staff often resulted from directly engaging with the KidsXpress team, or from teachers seeing improvements in their classroom due to trauma-informed approaches. It was often cited that key teachers who were passionate about trauma-informed teaching will be integral to maintaining this shift in school culture once the KidsXpress program has concluded.

“We have some very passionate people around trauma-informed practice….I think we’re in a good space with some really good voices to set up a team that can carry on the torch a little bit, when and if they [KidsXpress] eventually do go…I think all the staff are aware of its importance”. – School staff member

#### 3.5.4 Barriers to successful program implementation

Barriers to Implementation included a lack of sufficient KidsXpress staff and limited program days, which restricted access for students who needed support. Additionally, timetabling complexities and competing priorities were cited as significant barriers, along with gaining parental consent and willingness for participation.

“Not enough staff. There are many students who would benefit from participating in KidsXpress programs but are unable to due to time/staffing constraints.” – School Staff member.

“The complexities of school timetabling so that students don't miss literacy/numeracy sessions given the level of need in this area.” – School Staff member.

“Parent willingness to have their child/children participate.” – School Staff member.

This was reinforced by KidsXpress staff, who noted implementation challenges such as insufficient staffing, high demand, scheduling conflicts, and misconceptions about the program’s purpose, as illustrated by the following quote:

“Systemic issues, such as being constrained to the school timetable (can't run sessions during scripture, sport; can't run group program during ‘protected literacy’ time, etc.). Similarly, being asked to do something ‘urgently’, e.g., see a particular child going through a crisis, when we are not a crisis-intervention service and we do not currently have space in our timetable. … Another barrier is that the school staff do not seem very clear on what we do - e.g., one teacher expected us to teach social skills. We have been asked to do ‘more therapy, less play’, and to ‘talk’ to certain children. We have received feedback that a child will end the program having made no difference or experiencing ‘worse behaviours than before’ - I think this can sometimes be attributed to the teachers not observing the same things we observe or not measuring progress the same way we do.” – KidsXpress staff member.

Differences in cultural perspectives of mental health from the school community was a frequently cited barrier in program implementation. Participating schools had highly diverse school communities, and participants reported the need for cultural sensitivity when running therapy and psychoeducation sessions. Participants commented on a language barrier between KidsXpress staff and the school community, not necessarily limited to the meaning of words, but also the meaning of mental health and related jargon. Some participants reported that there can be stigma regarding mental health and help seeking among diverse school communities. This is further compounded by the concerns that the KidsXpress team are mandatory reporters, which was cited as a barrier for some families, and that a child with a mental health diagnosis may negatively affect citizenship for families who are not Australian citizens.

“In the cultures that are embedded in the school…there's quite a high caution on their privacy and customs and the way they live their life…So I think that causes a lot of reluctance, and I think it could be the taboo in mental health as well”. – KidsXpress staff member.

The difference between the strategic goals of the school and the therapeutic goals from the program was cited as an implementation barrier. Participants described this as a tension between the guidelines provided by the school or Department of Education, which were not always aligned with a trauma-informed approach. While school staff acknowledged that student wellbeing is integral to learning, they commented that their primary goals as educators is to teach, and not to provide a therapeutic space for their students. Participants commented that the goals of the school were driven by department and educational outcomes, whereas the goals of KidsXpress are emotional and psychosocial, resulting in a professional and philosophical barrier. Additionally, many participants commented that this barrier may not necessarily be overcome without top-down, systemic change.

“Just to take a completely trauma-informed approach, then I would not be following the department's policies, and my role is to follow the department's policies. So, we can, as much as possible, have a trauma-informed approach to managing challenging behaviours, but we do also have to implement department policy, and sometimes those things are not in alignment with each other”. – School staff member

Participants reported that some school staff have different or unrealistic expectations for students who attend therapy sessions. Participants commented that some teachers view KidsXpress as a program to “fix” challenging students, where these teachers can become frustrated if their referred students continue to misbehave. Further, it was reported that this misbelief can result in students with internalising mental health problems to fall through the cracks and not be referred on by teachers. KidsXpress staff reported sometimes being asked to improve a student's ability to sit still or perform academically, and they must explain that, while sometimes these behaviours may improve after therapy, this is not necessarily the service they provide.

“I think a lot of teachers think, “Oh, this kid has a behaviour, I’ll send them to KidsXpress”….We’ve had to do a lot of education around what we actually do…we’re actually not dealing with behaviours or academics or that kind of thing, but sometimes that's a great indirect benefit of the work”. – KidsXpress staff member.

“I think we could communicate more clearly about the purpose of the School Partnership. I think there are still lots of staff here who don't fully understand what we do and why.” – KidsXpress staff member.

“In the TIEC [trauma-informed education consultant] role I feel that more groundwork to make it really clear to staff that this role is to support the teachers (not students) to implement a trauma informed practice lens.” – KidsXpress staff member.

Participants commented that a minority of teachers are resistant to the services provided by KidsXpress. These teachers are often described as those who have been teaching for a long time and are not interested in changing their practice. Participants commented that these educators may feel intimidated by the presence of KidsXpress, or they may feel unsafe or insecure with external specialists coming into their classroom. Further, KidsXpress staff commented that school staff who don't have students in the program are often on the outskirts of their reach and require greater efforts to bring them onboard.

“There are definitely some strong barriers from certain teachers who made it really clear that they don't want us in the classroom or don't need coaching from us..usually those teachers are actually the ones that need support”. – KidsXpress staff member.

#### 3.5.5 Proposed solutions to barriers

School staff and KidsXpress staff made a number of recommendations to address the identified barriers, as detailed below.

It was commonly cited that there will always be the need in the participating schools to have more KidsXpress staff to meet school needs. All schools reported having a waitlist of students to access the program. Many participants commented that it would be helpful to have KidsXpress staff in school 4–5 days per week, suggesting that more therapists and/or consultants could share the workload and cover more days. Many participants suggested that having two consultants per school would allow for greater school community engagement and could facilitate change in school culture at a systemic level.

“At least at the minimum, I would advocate for another TIC [Trauma-informed consultant], because one can't do this work alone… just to have another person to be able to support teachers and educators of the school can make such a big difference”. – KidsXpress staff member.

Participants suggested that cultural competency training for KidsXpress staff could assist in supporting the school communities with diverse backgrounds. Some participants suggested this could take the form of workshops with the KidsXpress team, and others suggested that it would be helpful to have a member from that community to act as a cultural consultant. Participants commented that this role could be formal or informal, such as a teacher or parent providing cultural insights, or it could be a hired consultant advising how to facilitate and tailor the KidsXpress program for that community.

“If we could have more connection with cultural workers…better relationships with the local community who are able to speak into those gaps in knowledge and expectations…How good would it be if we had more access to these communities and more collaboration if, maybe, language and culture wasn't such a barrier?”. – KidsXpress staff member.

Some participants commented that ongoing communication and check-ins between the KidsXpress team and school staff and the executive team could help recruit greater support from the school community. Some school staff recommended that regular check-ins with the KidsXpress team could assist in creating shared goals to support their students referred into the program. Participants commented that experiential learning opportunities and wellbeing activities provided by KidsXpress for school staff, such as mock arts therapy sessions, could help teachers engage with the program when they may not have otherwise. Finally, KidsXpress staff commented that having consistent check-ins with the executive team would help ensure that the goals of KidsXpress are aligned with the goals of the school.

“Doing a [creative arts therapy] session with teachers…so teachers can see what's happening in those sessions and see the benefits of it…I don't know if a lot of people would understand it without experiencing themselves”. – School staff member

### 3.6 Maintenance

School staff generally felt that their school had improved trauma-informed practice since the establishment of the KidsXpress School Partnership (78.6% yes, 17.9% unsure, 3.6% no). However, they were less confident that their school would maintain this adherence to trauma-informed practice after the withdrawal of KidsXpress (59.1% yes, 34.8% unsure, 6.1% no). Open-ended survey responses revealed a strong desire for ongoing involvement of KidsXpress:

“Please don't go! The support is invaluable and irreplaceable.” – School staff member.

The majority of KidsXpress staff (*n* = 5, 71.4%) felt the school staff's perceptions of KidsXpress changed over time, while two participants (28.6%) were unsure. In contrast, most were unsure whether parents’ perceptions had changed over time (*n* = 4, 57.1%), while three participants (42.9%) thought they had changed. Open-ended survey responses suggested that changes in school staff perceptions were linked to increased understanding of expressive therapy and trauma-informed approaches, as well as growing trust and collaboration over time. Parent perceptions appeared to shift mainly when trust developed or when families had prior positive experiences with the program.

Half of KidsXpress staff (*n* = 3, 50%) felt their school had improved in trauma-informed practice since the implementation of the KX partnership, while one third were unsure. Of the half of participants who noticed an improvement, only one (33.3%) thought this improvement would be maintained after the withdrawal of the KidsXpress team.

## 4 Discussion

Early trauma can dramatically alter the trajectory of a child's emotional, cognitive, and behavioural development, inferring serious life-long consequences (7, 47, 48). The high prevalence of child trauma and adverse events experienced during childhood demands effective and early trauma support for children. Schools play a critical role in supporting children who have experienced trauma (14). This mixed-methods study represents a formal evaluation of the KidsXpress program, with a dual focus on implementation and effectiveness. The KidsXpress School Partnership Program was found to be an effective model for addressing trauma within this context, with benefits observed for all stakeholders. Despite many positive findings for the implementation of the KidsXpress School Partnership Program, a number of challenges were identified.

The KidsXpress School Partnership Program demonstrated good reach, with between 7%–57% of the student cohort referred for therapy. Schools with a longer time period since the establishment of the Partnership Program showed a greater rate of referrals, indicating successful partnerships take time to establish. Most referred students did receive therapy (73%–98%), but some were placed on a waitlist, and both teachers and KidsXpress staff highlighted unmet demand and that more students could have been referred if there was greater resourcing available. This pattern reflects findings from other trauma-informed school initiatives, which often report high demand and resource constraints as key implementation challenges (30). It suggests that while the program is accessible, additional resources are needed to expand its reach and ensure timely support for all students who require intervention.

Feedback from teachers and KidsXpress staff indicated strong perceived benefits of the program for both students and educators. For students, improvements included enhanced emotional regulation and coping strategies, increased confidence, greater willingness to seek help, and the ability to articulate feelings. These qualitative findings were supported by quantitative data showing significant improvements in student wellbeing scores, as well as school administrative data showing student attendance rates improved in four out of five schools. Teachers reported benefits such as increased understanding of trauma-informed practice, practical classroom strategies, and valuable mentoring and coaching from KidsXpress staff. Ratings of benefit for parents were lower, reflecting challenges in engaging families consistently. KidsXpress staff typically reported higher perceived benefit compared to school staff, potentially reflecting differences in understanding the goals of therapy or how progress is reflected. These findings are consistent with theoretical models underpinning trauma-informed and creative-expressive interventions. Observed improvements in emotional regulation, confidence, and relational engagement align with attachment-based and neurodevelopmental theories, which propose that restoration of safety and co-regulation is foundational to recovery from early adversity (34, 35). Importantly, understood within the context of the everyday classroom challenges associated with childhood trauma, the reported improvements in students’ emotional regulation, confidence, and relational engagement represent meaningful shifts in domains that directly impact classroom functioning. Similarly, increases in teacher confidence and understanding of trauma-informed practice suggest the program helped to equip teachers with the skills and sense of self-efficacy needed to effectively manage the day-to-day realities of working with children affected by trauma (11).

Despite overall improvements in student wellbeing, the magnitude of improvements differed between schools. However, it is unclear the extent to which this reflects real differences in the effectiveness of therapy. Indeed, for the newest partnership school, mean CORS scores at the start of therapy were very high (34 out of a possible 40) suggesting these students are either experiencing high levels of wellbeing prior to participating in the program or are not accurately reporting their wellbeing, choosing to inflate scores potentially due to a lack of trust or uncertainty in reporting lower scores. Thus, it remains unclear whether this reflects real differences in the effectiveness of therapy, differences in need between schools, or differences between schools as to whether students accurately report their wellbeing, particularly at the start of therapy. Students in schools with more established partnerships may have greater trust in KidsXpress therapists or the program by the time of their first therapy assessment if they have come into contact with these staff or heard about therapy from other students. The collection of detailed demographic data was intentionally limited to protect participant anonymity and encourage open reporting; however, this constrained the ability to examine differences across school or staff characteristics. Future research could unpack these findings further and understand how to ensure students feel comfortable reporting their needs early on in the therapeutic relationship.

Most participants agreed that the program fit well within the school context and was not disruptive to daily activities. The majority of teachers indicated they would recommend KidsXpress to other schools. Support from school executive teams emerged as a critical facilitator of successful implementation. Schools where leadership demonstrated commitment to trauma-informed principles and alignment with KidsXpress values were more likely to embed the program effectively. Executive endorsement created an environment where teachers felt encouraged to adopt trauma-informed approaches and collaborate with KidsXpress staff. Additionally, staff “champions”, who were passionate about trauma-informed practice were identified as key facilitators and critical in ensuring sustained trauma-aware practice after the withdrawal of the KidsXpress team. These findings are consistent with prior literature on implementation of school-based programs, which highlight that support at the organisational level is crucial (26, 28).

The current study highlights a number of implementation challenges to consider for others in the field. The most frequently cited challenges were insufficient staffing and cultural differences in perceptions of mental health. Across the board, participants reflected a desire for a greater number of KidsXpress staff to be embedded in the school. Schools serving culturally and linguistically diverse communities reported barriers such as stigma, language limitations, and concerns about mandatory reporting. Some parents expressed reluctance to engage due to fears that mental health diagnoses could affect immigration or citizenship status. KidsXpress staff also noted that psychoeducation workshops were not always culturally relevant, with some families preferring practical resources about navigating the school system. These challenges are consistent with literature emphasising the need for culturally responsive trauma-informed practices (49). Despite overall positive feedback, KidsXpress staff noted that teachers were not always clear on the roles of therapists and trauma-informed consultants or the goals of therapy. Misconceptions, such as viewing KidsXpress as a program to “fix” challenging students, sometimes led to frustration when behavioural issues persisted and may have led to under-referral of students with internalising difficulties. High staff turnover compounded this challenge, requiring repeated efforts to orient new teachers. Recommendations included stronger induction processes and resources to clarify program purpose and roles.

While school staff generally felt that trauma-informed practice had improved since the program's introduction, confidence in sustaining these changes after KidsXpress withdrawal was low. Similarly, KidsXpress staff were uncertain whether improvements would persist without ongoing support. This may reflect that the majority of schools involved in the School Partnership are within the first year of the partnership, which lasts three years, and thus are still in the initial stages of change. It is hoped that confidence in maintaining the improvements in trauma-informed practice would grow as it becomes more embedded in the actions and culture of the school. Nonetheless, the maintenance of mental health and public health interventions in schools generally has been identified as a substantial challenge (26, 50, 51), and underscores the need for strategies to embed trauma-informed approaches into the school ecosystem and ensure continuity beyond the program's duration (52).

To enhance implementation, several strategies could be considered. Increasing staffing and program days by expanding the number of therapists and trauma-informed consultants per school would help meet high demand and reduce waitlists. Strengthening communication with teachers through regular updates on student progress and timely sharing of practical strategies could improve classroom integration. Greater parent engagement is also critical, which may involve offering accessible workshops and culturally tailored resources, including translation and simplified materials. Providing cultural competency training or incorporating cultural consultants could address diverse community needs and reduce stigma around mental health. Clearer onboarding processes for school staff would ensure a shared understanding of program goals and the trauma-informed consultant role. Relationship-building with school staff and executives should remain a priority to foster trust and collaboration. Increasing flexibility in scheduling to minimise conflicts with core learning times and align with school calendars could further support integration. Finally, offering experiential learning opportunities, such as creative arts therapy demonstrations or wellbeing activities for teachers, may deepen understanding and engagement with the program.

### 4.1 Limitations

There are some limitations to this research that should be considered. Firstly, the schools involved in this research were a convenience sample of those who had already enrolled in the KidsXpress School Partnership program, and we did not have a control group, meaning we cannot attribute change to the KidsXpress Partnership Program. Secondly, participation was voluntary and not all staff participated, particularly in the qualitative interviews, with only three school staff participating. This may have introduced bias, with participants opting in having more extreme views (in either positive or negative direction), and there are likely viewpoints we were unable to capture with such a low sample. Relatedly, KidsXpress therapists and trauma-informed consultants were reporting on a program they are employed to implement. While the survey was anonymous, and interviews were conducted by independent researchers, it should be acknowledged that they have vested interests in the Partnership Program's success and this could have biased their responses. Third, the perspectives of caregivers and school executive teams were not included; however, this could be a valuable avenue for future research. Further, all schools differed in the time since establishment of the KidsXpress program, and thus many findings may reflect the amount of time elapsed, rather than differences in implementation of therapy or the partnership program itself. Particularly for whole-school indicators such as attendance, the reporting period was short, capturing 12-month changes in attendance. It is likely that capturing attendance over a longer time-frame would yield valuable insights, as schools gradually become more trauma aware, resulting in flow-on effects for indicators like attendance. Future research with longer follow-up periods would produce greater understanding of impact. Moreover, quantitative evaluation of program efficacy was limited to a single CORS measure; however, programs like KidsXpress are known to influence a broader range of student outcomes. Future research could explore these additional impacts by incorporating therapist notes or linking data with academic performance indicators such as grades and truancy. Finally, a randomised trial with a control group is important to understand whether change can be attributed to the KidsXpress School Partnership Program.

### 4.2 Implications for future research

Findings from this study highlight several important directions for future research. Longitudinal studies are needed to examine whether improvements in student wellbeing and trauma-informed practice are sustained over time, particularly following the withdrawal of external support. Randomised or quasi-experimental designs with comparison groups would strengthen causal inference regarding program effectiveness. Future research should also explore mechanisms of change, including how creative-expressive therapy and whole-school capacity-building interact to influence student outcomes. Greater inclusion of caregiver and school executive perspectives would provide a more comprehensive understanding of implementation and sustainability. Finally, research examining culturally responsive adaptations of trauma-informed school partnerships is warranted, particularly in culturally and linguistically diverse school communities.

## 5 Conclusions

This mixed-methods evaluation provides evidence that the KidsXpress School Partnership Program is a promising model for supporting children affected by trauma within primary school settings. Students who participated in creative-expressive therapy demonstrated significant improvements in wellbeing over the course of the program, supporting the early effectiveness of this developmentally appropriate, arts-based therapeutic approach. Importantly, these quantitative improvements were reinforced by consistent qualitative reports from school staff and KidsXpress personnel describing enhanced emotional regulation, confidence, coping, and help-seeking among participating students.

Beyond individual-level outcomes, this study highlights the value of a whole-school trauma-informed approach. Teachers reported increased understanding of trauma-informed practice, greater confidence in managing complex behaviours, and meaningful professional support through coaching and mentoring from KidsXpress staff. These findings underscore the importance of embedding specialist trauma expertise within schools to build staff capacity and promote systemic change.

At the same time, the study identified several implementation challenges that constrain program reach and sustainability, including insufficient staffing to meet high levels of demand, difficulties engaging families, particularly in culturally and linguistically diverse communities, and tensions between the priorities of the education system and therapeutic goals for the individual. While most participants perceived improvements in trauma-informed practice at their school, confidence in maintaining these changes following program withdrawal was limited, highlighting the ongoing challenge of sustaining mental health interventions in educational systems.

Collectively, these findings suggest that creative-expressive, trauma-informed school partnerships can deliver meaningful benefits for students and staff when implemented with strong leadership support, adequate resourcing, and clear communication regarding program goals and roles. Strategies to enhance sustainability, including expanded staffing models, culturally responsive engagement with families, clearer onboarding processes for school staff, and mechanisms to embed trauma-informed practices into school policies and routines, will be critical to maximising long-term impact.

## Data Availability

The datasets presented in this article are not readily available due to conditions of ethical approval. Some original data may be available upon request from the corresponding author. Requests to access the datasets should be directed to lucinda.grummitt@sydney.edu.au.
